# Bi-Gaussian analysis reveals distinct education-related alterations in spherical equivalent and axial length—results from the Gutenberg Health Study

**DOI:** 10.1007/s00417-024-06395-z

**Published:** 2024-03-06

**Authors:** Alica Hartmann, Stephanie Desirée Grabitz, Felix Mathias Wagner, Philipp Sebastian Wild, Martina Müller-Nurasyid, Karl Johannes Lackner, Manfred Elmar Beutel, Thomas Münzel, Oliver Tüscher, Jörn Markus Schattenberg, Norbert Pfeiffer, Alexander Karl-Georg Schuster

**Affiliations:** 1 Department of Ophthalmology, University Medical Center of the Johannes Gutenberg-University Mainz, Mainz, Germany; 2 Center for Thrombosis and Hemostasis, University Medical Center of the Johannes Gutenberg-University, Mainz, Germany; 3 German Center for Cardiovascular Research (DZHK), Partner Site Rhine-Main, Mainz, Germany; 4 Preventive Cardiology and Preventive Medicine – Department of Cardiology, University Medical Center of the Johannes Gutenberg-University, Mainz, Germany; 5 Institute of Molecular Biology (IMB), Mainz, Germany; 6 Institute for Medical Biometry, Epidemiology and Informatics (IMBEI), Mainz, Germany; 7 Institute of Clinical Chemistry and Laboratory Medicine, University Medical Center of the Johannes Gutenberg-University Mainz, Mainz, Germany; 8 Department of Psychosomatic Medicine and Psychotherapy, University Medical Center of the Johannes Gutenberg-University, Mainz, Germany; 9 Center for Cardiology, Cardiology I, University Medical Center of the Johannes Gutenberg-University Mainz, Mainz, Germany; 10 Clinic for Psychiatry and Psychotherapy, University Medical Center of the Johannes Gutenberg-University, Mainz, Germany; 11 Leibniz Institute for Resilience Research, Mainz, Germany; 12 Metabolic Liver Research Center, I. Department of Medicine, University Medical Center of the Johannes Gutenberg-University, Mainz, Germany

**Keywords:** Refraction, Myopia, Biometry, Mendelian randomization, Gaussian mixture model

## Abstract

**Purpose:**

The aim of this study is to investigate the distribution of spherical equivalent and axial length in the general population and to analyze the influence of education on spherical equivalent with a focus on ocular biometric parameters.

**Methods:**

The Gutenberg Health Study is a population-based cohort study in Mainz, Germany. Participants underwent comprehensive ophthalmologic examinations as part of the 5-year follow-up examination in 2012–2017 including genotyping. The spherical equivalent and axial length distributions were modeled with gaussian mixture models. Regression analysis (on person-individual level) was performed to analyze associations between biometric parameters and educational factors. Mendelian randomization analysis explored the causal effect between spherical equivalent, axial length, and education. Additionally, effect mediation analysis examined the link between spherical equivalent and education.

**Results:**

A total of 8532 study participants were included (median age: 57 years, 49% female). The distribution of spherical equivalent and axial length follows a bi-Gaussian function, partially explained by the length of education (i.e., < 11 years education vs. 11–20 years). Mendelian randomization indicated an effect of education on refractive error using a genetic risk score of education as an instrument variable (− 0.35 diopters per SD increase in the instrument, 95% CI, − 0.64–0.05, *p* = 0.02) and an effect of education on axial length (0.63 mm per SD increase in the instrument, 95% CI, 0.22–1.04, *p* = 0.003). Spherical equivalent, axial length and anterior chamber depth were associated with length of education in regression analyses. Mediation analysis revealed that the association between spherical equivalent and education is mainly driven (70%) by alteration in axial length.

**Conclusions:**

The distribution of axial length and spherical equivalent is represented by subgroups of the population (bi-Gaussian). This distribution can be partially explained by length of education. The impact of education on spherical equivalent is mainly driven by alteration in axial length.

**Supplementary Information:**

The online version contains supplementary material available at 10.1007/s00417-024-06395-z.

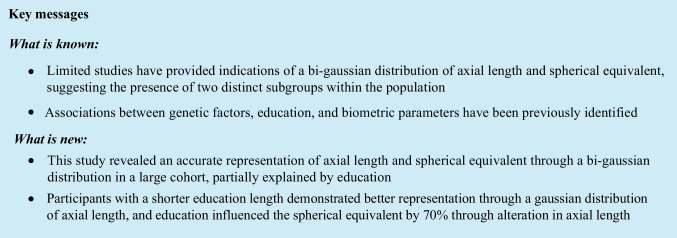



## Introduction

Most biological parameters under physiological circumstances follow a Gaussian distribution, as it was assumed for biometric eye parameters [1–3]. While there has been a consideration that refractive error follows a Gaussian distribution [4], it is essential to acknowledge early indications of a leptokurtic distribution dating back to 1864 [5]. In 2014, a European clinical study showed that the distribution of spherical equivalent resembles a bi-Gaussian distribution indicating a population with two separate subgroups. The same characteristic was seen for axial length, while anterior chamber depth (ACD) and lens power were better described as one Gaussian curve [1]. Understanding ocular biometry and its variation is essential to determine the power of intraocular lenses for achieving target refraction in cataract surgery [6], and to identify subjects at risk for ocular diseases.

Education and genetic factors have been shown to influence refractive error and axial length. Higher or longer education is correlated with longer axial length and myopia [7–11].

Mountjoy et al. [9] used Mendelian randomization technique to examine a possible causal relationship and reported a myopic increase of the refractive error of − 0.27 diopters per every additional year of education.

Genetic risk alleles for myopia were identified, which could lead to a higher risk of developing myopia [12–14]. In the past, the relationship between the genetic risk score and myopia showed a small effect in multivariable analysis [10]. An earlier study showed an impact of education on spherical equivalent independently of genetic risk. Still, it did not investigate the potential bi-Gaussian distribution and the underlying biometric parameters causing this association [10].

Thus, we aim to analyze the distribution of spherical equivalent and axial length in the general population in Germany. We model the impact of genetic parameters and education on axial length and spherical equivalent and also elaborate on the underlying ocular biometric parameters leading to the well-known association between higher education and myopia.

## Methods

### Procedure and study sample

The Gutenberg Health Study (GHS) is a population-based, prospective, observational single-center cohort study in the Rhine-Main-Region in Germany. The sample was equally stratified for sex, residence (urban or rural), and age-decade. A total of 12,423 individuals were re-examined at the 5-year follow-up (2012–2017) [15, 16].

### Ophthalmic parameters

The ocular biometry of the study participants was recorded using optical low-coherence reflectometry technology (LenStar900, Haag Streit, Switzerland) [15]. Patients were instructed to fixate on the center of the internal fixation target of the ocular biometer during the examination. The device performs three single measurements per examination and the parameters are averaged out of these measures. Ocular biometric measurements were excluded when they were likely to be invalid compared with other imaging modalities including Scheimpflug imaging (Pentacam HR, Oculus, Wetzlar, Germany). Objective refraction was measured with Humphrey Automated Refractor/Keratometer (HARK) 5991 [17].

### General parameters

Characteristics of the study population including age, sex, body height and body weight were surveyed. Other covariates included were socioeconomic status (SES) and education.

The degree of education was captured by three different variables:

Participants were asked to report their total duration of education in years (sum of years in school, vocational school and university; range: 0–20 years). Furthermore, the level of the completed educational training was queried by two additional questions:

1. What is your highest school-leaving qualification?
  - Secondary school (lowest level), total of 9 years of schooling (“Hauptschule”)
  - Secondary school (intermediate level), total of 10 years of schooling (“Realschule”)
  - High-school diploma/technical college certificate (“Abitur”)
2. What is your highest professional degree?
  - Vocational school (apprenticeship)
  - Vocational school, technician-, master school
  - University degree

### Genetic scores

An IlluminaOmniEURHD chip was used for genotyping. Imputation of the missing genotypes was performed using the imputation software Beagle using the 1000GP3 reference panel. The results were filtered and summarized as “allele dosage”, with imputation quality calculated using a ratio of observed and expected variance.

Three genetic risk scores were calculated: one for myopia, axial length and one for education. Single nucleotide polymorphisms (SNPs) known to be associated with refractive error were integrated, and 57 variants had been identified through the scientific literature [10, 14, 18], of which 4 were not in the panel of the phenotyping of the GHS (Appendix #1). For the axial length score, 46 SNPs were identified in the literature [18–20]. Of these, 21 were in the GHS panel (Appendix #2).

A previous study from the UK Biobank reported the calculation of a genetic risk score associated with education attainment/duration of education [9, 21]. Of the reported 74 genetic variants associated with education, 70 could be included in this analysis (Appendix #3).

### Inclusion and exclusion criteria

For the selection of study participants, only those with phakic lens status were included and participants who had cataract surgery were excluded.

### Statistical analysis

Descriptive analysis was conducted for primary and secondary variables. For categorical parameters, absolute and relative frequencies were computed. For continuous variables, mean and standard deviation was calculated for approximately normal-distributed data, otherwise median and interquartile range. The mean value from both eyes was used for the ophthalmic parameters, if available. Only phakic eyes were included [22].

Gaussian mixture models with the implementation of the expectation–maximization (EM) algorithm were used to analyze the distribution of spherical equivalent and axial length in the general population. Multivariate gaussian functions allow us to investigate whether the respective parameter follows a gaussian distribution or is better represented by subgroups in the population (bi- or multi-model-Gaussian distribution) [1]. The analysis considered the Akaike Information Criterion (AIC) and the Bayesian Information Criterion (BIC) values of the Gaussian mixture models. The AIC value indicates the likelihood of a model to estimate future values, with a good model characterized by a minimal AIC [23]. On the other hand, the BIC criterion captures the balance between the fit of the model and its complexity. A lower BIC value indicates a better fit of the model, striking a balance between accuracy and simplicity [24].

The genetic risk score for myopia was calculated by multiplying the frequency (0, 1, or 2) of the risk alleles with the effect estimate of the respective risk allele [25]. The same principle was applied to calculate the genetic score for education. However, in this case there were genes with negative coefficients. Here, three sum scores were calculated:

- Sum of SNPs with negative coefficients
- Sum of SNPs with positive coefficients
- Sum of SNPs

Linear regression analyses with generalized estimating equations were conducted to analyze the association between biometric parameters and education adjusted for age, sex and genetic risk score for myopia. The GEEMediate package in R was used to examine the natural direct effect on the mediation proportion on person-level using the mean value of both eyes.

As additionally analyses, possible sex differences were analyzed with linear regression models (with generalized estimating equations) stratified by sex. In addition, we conducted two sensitivity analyses. Firstly, we excluded all individuals over 70 years old to eliminate those with potential lens opacities, as this could impact the biometric parameters. The choice of the age threshold at 70 is based on a study by Stingl et al. [26]. In their investigation, a scatterplot reveals a quadratic relationship between a 5-year change in spherical equivalent (SE) and age. Specifically, there is a hyperopic shift observed between the ages of 44 and 70 years, and a myopic shift is evident at older ages. As a second sensitivity analysis, we excluded individuals with hyperopic refractive error that may be influenced by residual accommodation.

Bidirectional Mendelian randomization was performed to examine possible causal correlations between the duration of education and spherical equivalent, using the genetic risk score for education and the effect of spherical equivalent on duration of education, using the genetic risk score for myopia. The same principle was applied for axial length, involving a genetic score composed of SNPs associated with variations in axial length. Mendelian randomization (MR) is an analysis method which uses instrumental variables (IV). This analysis method allowed us to explore possible correlations by considering a genetically calculated risk. In MR analysis, alleles are randomly assigned; therefore, the analysis resembles a natural randomized control trial. This makes it less susceptible to bias than other analysis methods [27]. We conducted multiple MR methods: the inverse-variance weighted MR approach is most commonly used to examine the correlation between exposure and outcome in genome-wide association studies (GWAS). The approach is subject to the assumption that all SNPs are valid IVs. Further, the weighted MR method assumes that not all SNPs are valid IVs [28]. The MR-Egger approach is a sensitivity analysis to tests for directional pleiotropy; a low *p*-value demonstrates directional pleiotropy [29].

Data was processed with the statistical program R (version: 4.0.3 (2020–10-10)) with the packages clusterR, flexmix, GEEmediate, MendelianRandomization.

## Results

Overall, 8532 study participants were included. The median age of the study population was 57 years (age range: 40–80 years) and 49% were female. Table 1 shows the participant’s characteristics at the time point of the ophthalmologic examination (2012–2017). The biometric eye parameters of men were slightly larger/longer than women’s. Socioeconomic status and duration of education were also higher among men.

**Table 1:** Participants’ characteristics (*N* = 8532, data from the examination of the population-based Gutenberg Health Study 2012–2017). Values are presented as mean ± standard deviation unless stated otherwise

	Overall	Men	Women
Anthropometric data	8,532	4359	4173
Age (years), median (IQR), (min–max)	57 [49, 66] (40–80 years)	58 [50, 66] (40–80 years)	57 [49, 66] (40–80 years)
Weight (kg)	80.2 ± 16.87	87.85 ± 14.65	72.19 ± 15.24
Height (cm)	171 ± 10	177 ± 7	164 ± 7
BMI (kg/m^2^)	27.40 ± 5.00	27.92 ± 4.31	26.86 ± 5.59
Ophthalmic parameters			
Spherical equivalent, OD (diopter)	− 0.41 ± 2.51	–0.44 ± 2.44	–0.38 ± 2.58
Spherical equivalent, OS (diopter)	–0.42 ± 2.54	–0.45 ± 2.49	–0.39 ± 2.61
Axial Length, OD (mm)	23.74 ± 1.22	24.02 ± 1.19	23.44 ± 1.17
Axial Length, OS (mm)	23.71 ± 1.22	24.00 ± 1.22	23.41 ± 1.17
Anterior chamber depth, OD (mm)	3.25 ± 0.35	3.30 ± 0.36	3.20 ± 0.34
Anterior chamber depth, OS (mm)	3.24 ± 0.35	3.28 ± 0.35	3.19 ± 0.34
Corneal curvature, OD (mm)	7.84 ± 0.28	7.90 ± 0.28	7.78 ± 0.26
Corneal curvature, OS (mm)	7.83 ± 0.28	7.89 ± 0.28	7.77 ± 0.26
Lens thickness, OD (mm)	4.35 ± 0.37	4.37 ± 0.38	4.34 ± 0.35
Lens thickness, OS (mm)	4.42 ± 0.36	4.44 ± 0.37	4.39 ± 0.35
White-to-white, OD (mm)	12.21 ± 0.43	12.29 ± 0.44	12.12 ± 0.41
White-to-white, OS (mm)	12.21 ± 0.44	12.29 ± 0.45	12.13 ± 0.41
Socio-economic data			
Socio-economic status (SES)	13.30 ± 4.38	13.91 ± 4.41	12.66 ± 4.27
Duration of education (years)	13.05 ± 2.09	13.26 ± 2.15	12.84 ± 2
Completion of training			
Secondary school (lowest level), total of 9 years of schooling), Hauptschule, *n* (%)	2727 (32.1)	1422 (32.8)	1305 (31.4)
Secondary school (intermediate level), total of 10 years of schooling, Realschule, *n* (%)	2174 (25.6)	871 (20.1)	1303 (31.3)
Technical college certificate, *n* (%)	901 (10.6)	591 (13.6)	310 (7.5)
High-school diploma (Abitur), *n* (%)	2652 (31.2)	1426 (32.9)	1226 (29.5)
Other degree/no degree	40 (0.5)	26 (0.6)	14 (0.3)
Training degree			
Vocational school (apprenticeship), *n* (%)	3836 (45.1)	1622 (37.4)	2214 (53.2)
Vocational school, technician-, master school, *n* (%)	1300 (15.3)	815 (18.8)	485 (11.7)
College of applied science, *n* (%)	1098 (13.0)	733 (16.9)	365 (8.8)
University degree, *n* (%)	1725 (20.3)	987 (22.8)	738 (17.7)
Other degree/no degree (%)	535 (6.3)	179 (4.2)	356 (8.6)

### Gaussian mixture models

**Fig. 1:**
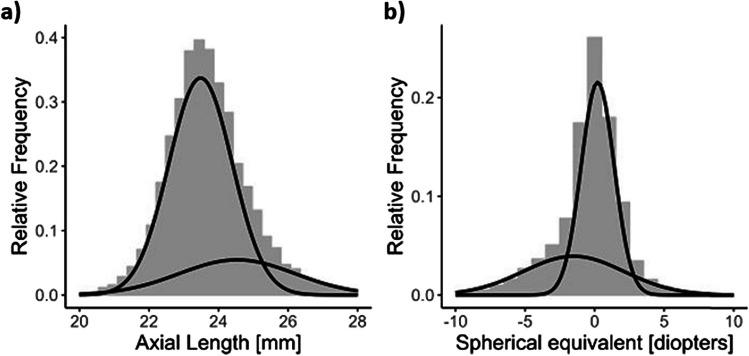
Bi-Gaussian model: **a** axial length, **b** spherical equivalent. Results from the population-based Gutenberg Health Study (*n* = 8532; 2012–2017)

The Gaussian mixture models showed that a bi-Gaussian function could better represent the distribution of some biometric parameters in the population. Two subgroups better represent the distribution of axial length and the spherical equivalent, while one Gaussian curve best describes the distribution of anterior chamber depth and corneal curvature (Fig. 1). The AIC and BIC values of the bi-Gaussian models were lower than those with one curve, indicating a better population representation of the population by two subgroups.

**Fig. 2:**
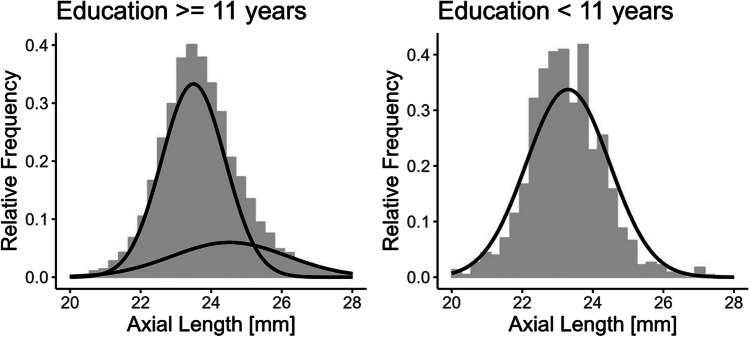
Gaussian and bi-Gaussian model. Axial length stratified on educational length. Results from the population-based Gutenberg Health Study (*n* = 8532; 2012–2017)

**Fig. 3:**
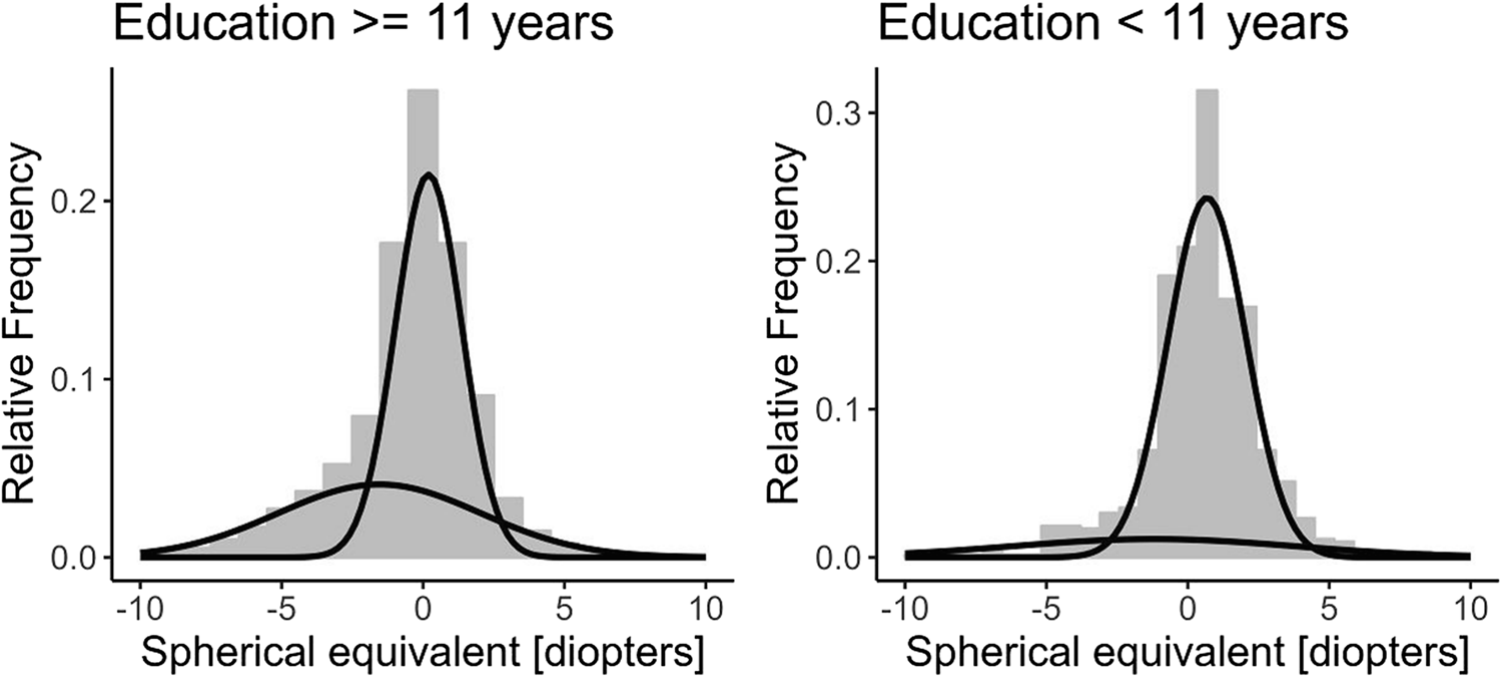
Gaussian and bi-Gaussian model. Spherical equivalent stratified on educational length. Results from the population-based Gutenberg Health Study (*n* = 8532; 2012–2017)

When stratifying the study sample for persons with 0–10 years and 11 + years, there was a Gaussian distribution in subjects for persons with a shorter duration of education for axial length (Fig. 2). However, with a longer duration of education the axial length showed a bi-Gaussian distribution. Additionally, the spherical equivalent still showed a flattened second curve with shorter duration of education but the histogram is less left skewed (Fig. 3).

### Associations between refractive error and education

Linear regression analysis using generalized estimating equations (GEE) showed an association between axial length and duration of education. The association mentioned above were also found for anterior chamber depth and spherical equivalent. Results highlight the spherical equivalent decreases with each additional year of education. Axial length becomes longer with each additional year of education. This is also the case for anterior chamber depth, while lens thickness, corneal curvature and white-to-white distance did not reveal an association with education (Table 2). In the conducted sensitivity analyses, where individuals over 70 were excluded in one instance and those with a hyperopic refractive error were excluded in the second, comparable results were observed.

**Table 2:** Association analysis between ocular biometric parameters and duration of education, data from the German population-based Gutenberg Health Study (2012–2017). Linear regression analyses were performed using GEE estimations, adjusted for age, sex, and genetic risk score for myopia

	Year of education		
	Estimate	95%-CI	*p*
Spherical equivalent	− 0.10	[− 0.13– − 0.08]	< 0.001
Axial length	0.06	[0.05–0.07]	< 0.001
Corneal curvature	0.002	[0.00–0.01]	0.12
Anterior chamber depth	0.01	[0.00–0.01]	< 0.001
Lens thickness	− 0.002	[− 0.01–0.00]	0.08

We additionally stratified the regression models by sex and found one sex-related difference (Supplemental Table 1). Corneal curvature and duration of education is associated only in female participants. Apart from this result, the results fit the regression analysis for all participants.

To investigate whether the association between duration of education and spherical equivalent is mediated by alteration of axial length, a further model was applied. The marginal model (model without the mediator) showed a significant relationship between the spherical equivalent and duration of education (beta =  − 0.18, *p* < 0.001). The conditional model (with the mediator axial length) demonstrated that the effect estimate decreases by 70%, the remaining effect was still significant (beta =  − 0.05, *p* < 0.001).

### Mendelian randomization

**Fig. 4:**
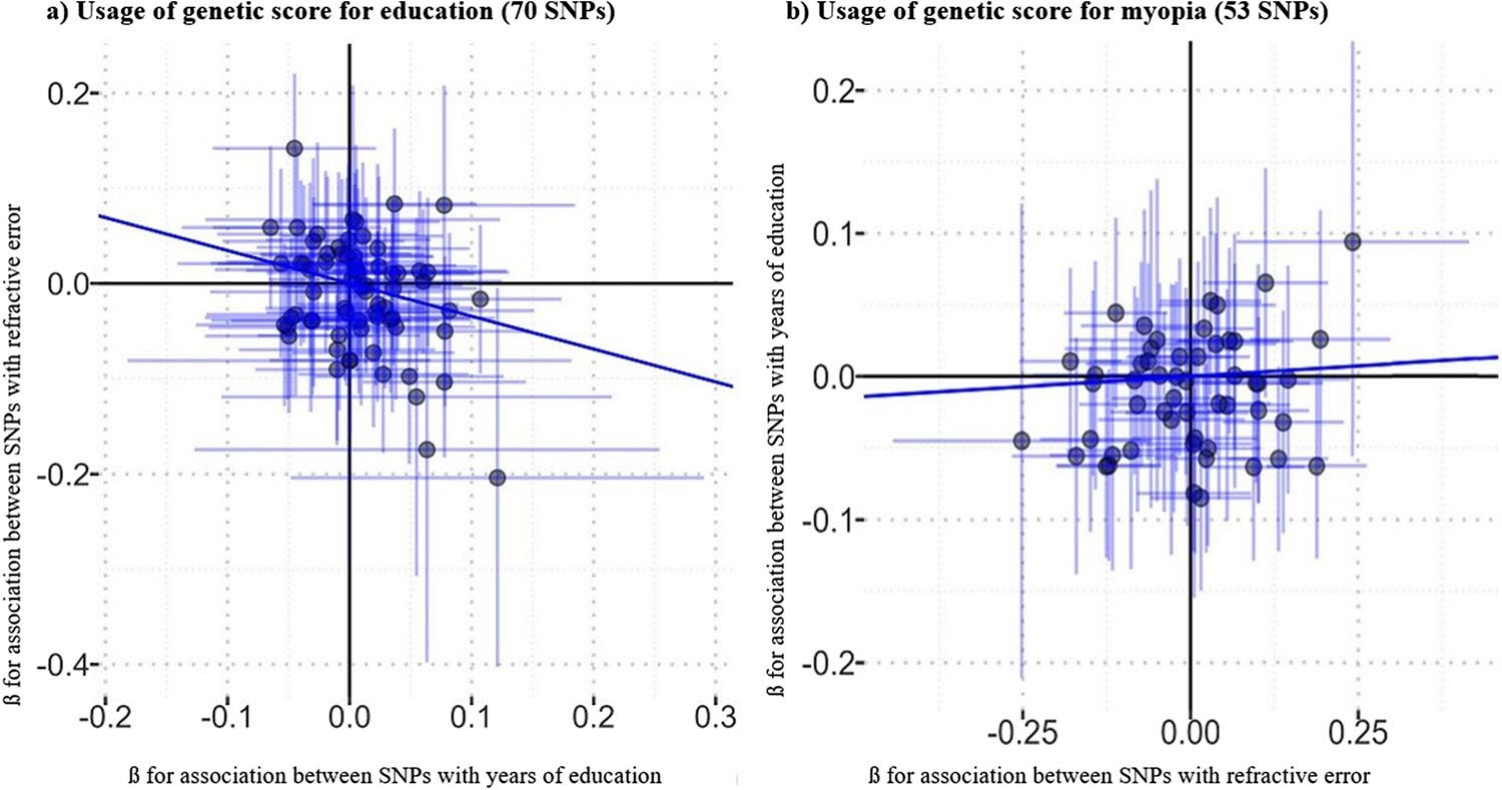
Results of bidirectional Mendelian Randomization, standard inverse-variance weighted method. Data from the German population-based Gutenberg Health Study (2012–2017). Regression line and standard errors (shaded area) fitted using robust linear regression. Whiskers represent 95% confidence intervals

The inverse-variance weighted (IVW) MR indicated that there might be a causal effect of education years on refractive error using the genetic score for education (− 0.35 diopters per SD increase in the instrument, 95% CI, − 0.64–0.05, *p* = 0.02; Table 3, Fig. 4a). Furthermore, there was no evidence of a causal effect of refractive error on education, based on the risk score for myopia (Table 3, Fig. 4b). The MR-Egger intercept test showed no average directional pleiotropy in either models (*p* = 0.09 and *p* = 0.25).

**Table 3:** Results of bidirectional Mendelian randomization (MR) for refractive error and axial length. Data from the German population-based Gutenberg Health Study (2012–2017)

MR Analysis for education years on refractive error				MR Analysis for education years on axial length		
	Estimate	95% CI	*p*-value	Estimate	95% CI	*p*-value
Simple median	− 0.36	[− 0.80–0.08]	0.11	0.80	[0.18–1.42]	0.01
Weighted median	− 0.18	[− 0.62–0.27]	0.44	0.92	[0.28–1.56]	0.01
IVW	− 0.35	[− 0.64– − 0.05]	0.02	0.63	[0.22–1.04]	0.003
MR-Egger	− 0.42	[− 0.89–0.06]	0.09	0.61	[− 0.05–1.23]	0.07
Intercept	0.004	[− 0.02–0.02]	0.71	0.001	[− 0.01–0.01]	0.93
MR Analysis for refractive error on years of education				MR analysis for axial length on years of education		
Simple median	0.01	[− 0.21–0.23]	0.94	0.10	[− 0.54–0.73]	0.77
Weighted median	− 0.003	[− 0.19–0.19]	0.97	0.26	[− 0.29–0.81]	0.35
IVW	0.03	[− 0.09–0.15]	0.65	0.19	[− 0.29–0.67]	0.45
MR-Egger	0.12	[− 0.09–0.33]	0.25	0.06	[− 0.68–0.80]	0.88
Intercept	− 0.01	[− 0.03–0.01]	0.27	0.01	[− 0.02–0.04]	0.65

Figure 4 shows the findings of the IVW approach. 5 of 70 variants associated with duration of education were significantly associated with higher levels of myopia in our study population (more negative SE, Fig. 4a). A total of 21 of 53 variants associated with myopia were not associated with longer duration of education (Fig. 4b).

An effect of duration of education on axial length was also demonstrated using the genetic score for education (0.63 mm per SD increase in the instrument, 95% CI, 0.22–1.04, *p* = 0.003). There was no significant effect of axial length on education when using the genetic score for axial length. No directional pleiotropy was demonstrated in both models (*p* = 0.07 and *p* = 0.88).

## Discussion

This study investigated the distribution of spherical equivalent and axial length in the general population. The impact of education on the spherical equivalent and axial length as biometric parameters was analyzed. Our results highlight that the spherical equivalent and axial length distribution follow a bi-Gaussian distribution. The distribution can be partially explained by the length of education (i.e., < 11 years of education vs. 11–20 years). Mendelian randomization showed that spherical equivalent and axial length is influenced by duration of education.

In 2013, Rozema et al. investigated the distribution of spherical equivalent in more detail and further analyzed the distribution of axial length. This study supports the finding of a bi-Gaussian distribution for the axial length and spherical equivalent [1]; nevertheless, the reason for this bi-Gaussian distribution remained in the most studies unexplained. Flitcroft’s review [30] suggests that human myopia may result from a failure of homeostasis, particularly emmetropization—a natural process where the eye tends to grow towards an optimal state in early life [31, 32]. Homeostatic failures can lead to refractive errors, increasing variability. Acknowledging myopia as a homeostatic failure implies diverse causes, allowing identification of subgroups responsive to specific influences, genes [33], or treatments [30].

Our study showed an association between spherical equivalent and duration of education in linear regression analysis and through Mendelian randomization technique. This is in line with a recent study [9] investigating the direction of causality in the relationship of myopia and education using Mendelian randomization technique. While the authors concluded that myopia did not influence educational level, a myopic shift of − 0.27 diopters was observed for each additional year of education [9]. This finding is higher than our result of -0.10 diopters for each additional year of education in the regression analysis.

Furthermore, the stratification of the regression models by sex showed one relevant difference. The association between corneal curvature and duration of education was only visible in female participants. It is important to note that, overall, no consistent relationship between education duration and corneal curvature has been observed, and other studies have also failed to demonstrate a significant association between education and corneal curvature [34]. This sex difference could therefore result from different sample characteristics, or chance finding.

In 2016, Mirshahi et al. analyzed the relationship between myopia and cognitive performance. Cognitive performance was assessed using the Tower of London Test. The findings of the linear mixed model indicated that the length of education influences on myopia (beta =  − 0.14, *p* < 0.001). In contrast, there was no relationship between cognitive performance and myopia (beta =  − 0.0017, *p* = 0.21) [35].

Previous studies have demonstrated a correlation between axial length and myopia with a higher level of education [7–11, 36]. This study provides evidence that the correlation between myopia and education may be attributed, in part, to a bi-Gaussian distribution of axial length.

In addition, our analysis showed that axial length increased with each year of education.

Education contributes to increased hours of near work through reading and writing. Studies showed that a longer duration of near work and a small distance between the eyes and the objects viewed are associated with an increased risk of myopia [37–39]. Data from the “British Twins Early Development Study (TEDS)” [40], examined twins aged 5 to 12 years in 1996. This study indicated that near work and screen time were associated with a higher risk of myopia in childhood. Near work requires the eye to adjust to the varying distances of the objects constantly. One possible reason for this could be when individuals engage in prolonged near activities, such as reading, this may lead to a blurred retinal image in the mid-periphery known as peripheral hyperopic defocus. Studies in animals have indicated that peripheral hyperopic defocus stimulate the growth of the eye [39, 41, 42].

Previous studies have demonstrated an association between myopia and environmental and genetic factors. Studies of identical twins and families showed a strong hereditary component to myopia [40, 43]. Several genes have been identified that influence axial length and spherical equivalent [18, 20]. Possible environmental risk factors for myopia, such as using electronic devices, television, or computer [44, 45], are addressed. Outdoor time and light exposure are myopia protective factors [46]. Sherwin et al. reported 2% reduced odds of myopia per additional hour per week spent outside [47].

The change in refractive error is directly linked to the previously identified risk factors: The risk factors lead to a change in biometric parameters for instance axial length [45]. In the retina, dopamine is an important neurotransmitter responsible for various functions: it is relevant for the creation of visual signals as well as refractive development [48]. Brighter light leads to a release of dopamine attenuating axial length growth. Some of the risk factors may arise due to competing activities: increased media use is associated with less time outdoors, thus resulting in less exposure of the eye to bright light [46].

## Strengths and limitations

This study analyzed data from a large population-based representative sample and contributed to a better understanding of the distribution of spherical equivalent and axial length in the general population. Education as an underlying factor for the occurred distributions could be demonstrated. Compared to other studies on this topic, the large study population offers the possibility of obtaining representative findings. However, some limitations in our study need to be considered. First, the included GHS subjects mainly consist of Caucasian origin. Therefore, the results cannot be generalized to other ethnicities. A second limitation is that no data on the previous outdoor activity of the study population and no parameters on light exposure, especially during childhood, adolescence and early adulthood were collected. The influence of outdoor activity separate light exposure on myopia has been proven [49–51]. Thus, adjusting for outdoor activity in the regression analysis was impossible. After age 35, a further myopic shift is less likely as a recent publication has shown [26], and there is no association between type of occupation and refractive error.

## Conclusion

In conclusion, this study demonstrated that the distribution of axial length and spherical equivalent is better represented by two subgroups (bi-Gaussian) in a population-based study. Stratification of the study population by duration of education (education 0–10 years vs. education 11–20 years) showed that for a short duration of education, the distribution of axial length follows the physiological Gaussian distribution. The distribution of spherical equivalent follows nearly a Gaussian distribution. Myopic spherical equivalent, longer axial length and deeper anterior chamber depth were associated with duration of education, while corneal curvature and lens thickness were not. Mediation analysis showed that about 70% of the effect of education on spherical equivalent is due to elongation in axial length.

### Supplementary Information

Below is the link to the electronic supplementary material.

Supplementary file1 (PDF 1079 KB)
