# Supplementary material for: Bi-Gaussian analysis reveals distinct education-related alterations in spherical equivalent and axial length—results from the Gutenberg Health Study
Source: Graefes Arch Clin Exp Ophthalmol. 2024 Mar 6;262(6):1819–28. doi: 10.1007/s00417-024-06395-z (PMC11106185; doi:10.1007/s00417-024-06395-z)
Supplement: Supplementary file 1 — Supplementary file1 (PDF 1079 KB) [file 417_2024_6395_MOESM1_ESM.pdf]

Appendix 1

**Genes associated with refractive error** (DOI: 10.1016/j.ophtha.2014.04.017)	**SNP**	**Position**	**chr**	**allele**
ANTXR2	rs11939401	80818417	4	C/T
B4GALNT2	rs9902755	47220726	17	C/T
BICC1	rs4245599	60365755	10	G/A
BMP3	rs5022942	81959966	4	G/A
BMP4	chr14:54413001	54413001	14	G/C
DLG2	rs2155413	84634790	11	C/A
DLX1	rs17428076	172851936	2	C/G
EHBP1L1	chr11:65348347	65348347	11	G/A
GOLGA8B/ GJD2	rs524952	35005886	15	T/A
GPR25	rs6702767	200844547	1	G/A
KCNMA1	rs6480859	79081948	10	C/T
KCNQ5	rs7744813	73643289	6	A/C
LAMA2	rs12193446	129820038	6	A/G
LRFN5	rs61988414	42313443	14	A/G
LRRC4C	rs1381566	40149607	11	T/G
MYO1D/TMEM98	rs10512441	31239645	17	C/T
NPLOC4	chr17:79585492	79585492	17	G/A
PRSS56	rs1550094	233385396	2	A/G
PABPCP2	rs17412774	146773948	2	A/C
PDE11A	rs17400325	178565913	2	T/C
PZP	rs6487748	rs6487748	12	A/G
QKI	rs9365619	164251746	6	C/A
RASGRF1	rs28412916	79378167	15	A/C
RBFOX1	rs17648524	7459683	16	G/C
RDH5	rs3138142	56115585	12	C/T
RGR	rs745480	85986554	10	C/G
SETMAR	rs1843303	4185124	3	T/C
SFRP1	rs2137277	40734662	8	A/G
SH3GL2/ADAMTSL1	rs10963578	18338649	9	G/A
SHISA6	rs2908972	11407259	17	T/A
TCF7L2	rs4367880	114795256	10	G/C
TOX/CA8	chr8:60178580	60178580	8	C/G
TJP2	rs11145746	71834380	9	G/A
ZBTB38	rs13091182	141133960	3	G/A
ZIC2	rs4291789	100672921	13	C/G

**Genes associated with refractive error** (DOI:https://doi.org/10.1038/s42003-020-0802-y)	**SNP/rsid**	**Position**	**chr**	**allele**
ADAMTS20	rs11181913	43574200	12	A/G
CASC15	rs9366426	22064639	6	T/C
CHRND	rs2245601	233390937	2	A/G
CWC27/ADAMTS6	rs11740254	64319640	5	T/C
FBN1	rs9806595	48755168	15	T/C
FGF9	rs9506725a	22314146	13	T/C
HUS1	rs12702376	47775053	7	C/G
IGFBP5	rs1548942	217619036	2	T/C
INTS6	rs7327381	52006645	13	T/C
OFCC1	rs67612840 6	10028826	6	A/T
KAZALD1	rs807037	102824349	10	C/G
RNLS	rs166976	90024599	10	A/G
RP11-91P17.1	rs7004112	78941331	8	T/G
THBS4	rs13180294	79360175	5	A/G
WNT7B	rs200329677	45,973,898	22	C

**Genes associated with refractive error** (DOI: 10.1038/ng.2554)	**SNP**	**Position**	**chr**	**allele**
BMP2	rs235770	6761764	20	T/C
GJD2	rs524952	35005885	15	A/T
GRIA4	rs11601239	105061808	11	C/G
LOC100506035	rs9307551	80530670	4	A/C
PTPRR	rs12229663	71249995	12	G/A
SIX6	rs1254319	60903756	14	A/G
THBS4	rs13180294	79360175	5	A/G

Appendix 2

**SNPs associated with axial length** (https://www.sciencedirect.com/science/article/pii/S2666914522000021#appsec1)	**Nearest gene**
rs16959560	LINC02252wGJD2
rs151278468	ANKFN1wNOG
rs141313179	VIPR2
rs10453459	WNT7B
rs7936359	LRRC4C
rs13380109	RASGRF1
rs77311538	PRSS56
rs4823003	ZNRF3
rs10459508	BMP4wCDKN3
rs4889024	MAFTRR
rs7744813	KCNQ5
rs3138142	RDH5
rs11204213	RBP3
rs16890057	ZMAT4
rs3848363	AXIN1
rs36005291	LOC100505501wCA8
rs7651194	ELP6
rs146314970	BMP2
rs11325378	ZNF543
rs58353542	LOC102724511w LOC154449
rs3741210	IGF2-AS
rs147732642	GSX2wPDGFRA
rs965480	HMG20Aw LOC101929457
rs72609833	SNTB1
rs7902218	CNNM2
rs35305813	GLRA1
rs60806750	MIR548ADw CRIM1-DT

**SNPs associated with axial length** (https://pubmed.ncbi.nlm.nih.gov/22685421/)	**Nearest gene**
rs4373767	ZC3H11B
rs10779363	ZC3H11B
rs7544369	ZC3H11B
rs4428898	ZC3H11B
rs4557020	SPTBN1
rs282544	PARP8
rs1137	SEMA4F
rs2404958	PARP8
rs10735496	ZC3H11B
rs4671938	SPTBN1
rs32396	PARP8
rs12055210	PARP8
rs11954386	PARP8

**SNPs associated with axial length** (https://pubmed.ncbi.nlm.nih.gov/24144296/)	**Nearest gene**
rs4074961	*RSPO1* (MIM 609595)
rs994767	*ZC3H11B*
rs9811920	*C3orf26*
rs12193446	*LAMA2* (MIM 156225)
rs11073058	*GJD2* (MIM 607058)
rs12321	*ZNRF3* (MIM 612062)

Appendix 3

Data from the UK Biobank: association between SNPs and education in the pooled-sex *EduYears* meta-analysis (DOI: 10.1038/nature17671)

				Pooled					Males		Females	
SNP	Chr	Position	Allele 1	Frequency Allele 1	Effect size	*P*-value	Heterogeneity I^2	Heterogeneity *P*-value	Effect size	*P*-value	Effect size	*P*-value
rs301800	1	8490603	T	0.18	0.019	1.79E-08	0.0	0.516	0.022	2.19E-06	0.022	2.19E-06
rs11210860	1	43982527	A	0.37	0.017	2.36E-10	0.0	0.796	0.019	2.83E-07	0.019	2.83E-07
rs34305371	1	72733610	A	0.09	0.035	3.76E-14	22.5	0.071	0.040	2.85E-10	0.040	2.85E-10
rs2568955	1	72762169	T	0.24	-0.017	1.80E-08	0.0	0.841	-0.020	1.11E-06	-0.020	1.11E-06
rs1008078	1	91189731	T	0.41	-0.016	6.01E-10	21.7	0.070	-0.016	7.50E-06	-0.016	7.50E-06
rs11588857	1	204587047	A	0.21	0.020	5.27E-10	0.8	0.459	0.019	5.37E-06	0.019	5.37E-06
rs1777827	1	211613114	A	0.59	0.015	1.55E-08	3.4	0.400	0.011	1.32E-03	0.011	1.32E-03
rs2992632	1	243503764	A	0.72	0.017	8.23E-09	0.0	0.780	0.017	2.00E-05	0.017	2.00E-05
rs76076331	2	10977585	T	0.15	0.020	3.63E-08	0.0	0.735	0.024	1.76E-06	0.024	1.76E-06
rs11689269	2	15621917	C	0.33	0.016	1.28E-08	0.0	0.484	0.017	5.78E-06	0.017	5.78E-06
rs1606974	2	51873599	A	0.12	0.022	2.80E-08	19.8	0.092	0.016	2.66E-03	0.016	2.66E-03
rs11690172	2	57387094	A	0.59	0.015	1.99E-08	16.2	0.141	0.018	5.33E-07	0.018	5.33E-07
rs2457660	2	60757419	T	0.64	-0.017	7.11E-10	0.0	0.821	-0.013	3.68E-04	-0.013	3.68E-04
rs114598875	2	60976384	A	0.84	-0.020	2.41E-08	0.0	0.991	-0.022	3.14E-06	-0.022	3.14E-06
rs10496091	2	61482261	A	0.29	-0.018	5.62E-10	10.1	0.252	-0.014	2.42E-04	-0.014	2.42E-04
rs13402908	2	100333377	T	0.46	-0.018	1.70E-11	0.0	0.992	-0.020	1.37E-08	-0.020	1.37E-08
rs4851251	2	100753490	T	0.27	-0.017	1.91E-08	0.7	0.460	-0.016	6.85E-05	-0.016	6.85E-05
rs12987662	2	100821548	A	0.39	0.027	2.69E-24	19.9	0.090	0.028	1.17E-14	0.028	1.17E-14

rs17824247	2	144152539	T	0.59	-0.016	2.77E-09	0.0	0.571	-0.014	1.20E-04	-0.014	1.20E-04
rs16845580	2	161920884	T	0.63	0.016	2.65E-09	0.0	0.872	0.016	1.18E-05	0.016	1.18E-05
rs4500960	2	162818621	T	0.46	-0.016	3.75E-10	0.0	0.591	-0.020	1.89E-08	-0.020	1.89E-08
rs6739979	2	193731929	T	0.63	-0.015	4.70E-08	0.0	0.586	-0.010	7.02E-03	-0.010	7.02E-03
rs2245901	2	194296294	A	0.40	-0.016	4.54E-09	0.0	0.863	-0.014	8.63E-05	-0.014	8.63E-05
rs55830725	2	237056854	A	0.17	-0.022	5.37E-10	3.3	0.403	-0.019	5.21E-05	-0.019	5.21E-05
rs35761247	3	48623124	A	0.05	0.034	3.82E-08	0.0	0.488	0.033	7.86E-05	0.033	7.86E-05
rs62259535	3	48939052	A	0.96	0.048	2.63E-09	3.2	0.415	0.048	1.11E-05	0.048	1.11E-05
rs148734725	3	49406708	A	0.32	0.025	1.36E-18	3.8	0.391	0.028	1.12E-13	0.028	1.12E-13
rs11712056	3	49914397	T	0.57	0.024	3.30E-19	40.3	0.001	0.025	6.02E-13	0.025	6.02E-13
rs112634398	3	50075494	A	0.95	0.036	4.61E-08	0.0	0.878	0.041	3.96E-06	0.041	3.96E-06
rs62263923	3	85674790	A	0.64	-0.016	7.01E-09	15.4	0.154	-0.012	6.62E-04	-0.012	6.62E-04
rs6799130	3	160847801	C	0.52	-0.015	2.82E-08	25.0	0.041	-0.013	3.00E-04	-0.013	3.00E-04
rs12646808	4	3249828	T	0.66	0.016	4.00E-08	16.1	0.148	0.018	4.06E-06	0.018	4.06E-06
rs2610986	4	18037231	T	0.67	-0.016	2.01E-08	3.6	0.398	-0.018	4.17E-06	-0.018	4.17E-06
rs34072092	4	28801221	T	0.90	0.024	3.91E-08	5.0	0.364	0.017	3.28E-03	0.017	3.28E-03
rs3101246	4	42649935	T	0.60	-0.015	1.43E-08	5.5	0.354	-0.022	1.96E-09	-0.022	1.96E-09
rs4863692	4	140764124	T	0.31	0.018	1.56E-10	4.6	0.371	0.024	1.66E-10	0.024	1.66E-10
rs4493682	5	45188024	C	0.17	0.019	3.32E-08	0.0	0.959	0.026	3.71E-08	0.026	3.71E-08
rs2964197	5	57535206	T	0.50	0.015	3.02E-08	6.7	0.329	0.012	4.19E-04	0.012	4.19E-04
rs61160187	5	60111579	A	0.61	-0.017	3.49E-10	17.2	0.129	-0.018	8.38E-07	-0.018	8.38E-07
rs324886	5	87896602	T	0.39	-0.015	1.91E-08	0.0	0.485	-0.019	1.77E-07	-0.019	1.77E-07

rs10061788	5	87934707	A	0.18	0.021	2.46E-09	0.0	0.545	0.020	1.73E-05	0.020	1.73E-05
rs2431108	5	103947968	T	0.68	0.016	5.27E-09	0.0	0.534	0.014	1.98E-04	0.014	1.98E-04
rs1402025	5	113987898	T	0.78	0.017	3.42E-08	11.4	0.227	0.015	4.75E-04	0.015	4.75E-04
rs62379838	5	120102028	T	0.69	0.016	3.30E-08	0.0	0.811	0.021	4.83E-08	0.021	4.83E-08
rs56231335	6	98187291	T	0.67	-0.017	2.07E-09	4.0	0.387	-0.020	1.11E-07	-0.020	1.11E-07
rs9320913	6	98584733	A	0.48	0.024	2.46E-19	0.0	0.717	0.027	3.25E-14	0.027	3.25E-14
rs7767938	6	153367613	T	0.75	0.017	2.44E-08	0.0	0.662	0.018	1.25E-05	0.018	1.25E-05
rs2615691	7	23402104	A	0.04	-0.037	4.71E-08	0.0	0.961	-0.041	1.04E-05	-0.041	1.04E-05
rs12531458	7	39090698	A	0.51	0.014	3.11E-08	7.9	0.300	0.011	1.34E-03	0.011	1.34E-03
rs12671937	7	92654365	A	0.53	0.016	9.15E-10	0.0	0.763	0.021	9.51E-09	0.021	9.51E-09
rs113520408	7	128402782	A	0.27	0.017	1.97E-08	9.3	0.270	0.019	1.60E-06	0.019	1.60E-06
rs17167170	7	133302345	A	0.80	0.020	1.14E-09	0.0	0.818	0.022	4.25E-07	0.022	4.25E-07
rs11768238	7	135227513	A	0.34	-0.017	9.90E-10	0.0	0.802	-0.019	5.12E-07	-0.019	5.12E-07
rs12682297	8	145712860	A	0.46	-0.016	3.93E-09	4.7	0.372	-0.015	1.25E-05	-0.015	1.25E-05
rs1871109	9	1746016	T	0.55	-0.016	4.35E-10	6.3	0.335	-0.015	1.48E-05	-0.015	1.48E-05
rs13294439	9	23358875	A	0.59	-0.023	2.20E-17	6.7	0.328	-0.022	3.93E-09	-0.022	3.93E-09
rs895606	9	88003668	A	0.45	0.015	2.25E-08	14.2	0.176	0.018	1.44E-07	0.018	1.44E-07
rs7854982	9	124644562	T	0.46	-0.015	1.29E-08	0.0	0.902	-0.020	1.42E-08	-0.020	1.42E-08
rs11191193	10	103802408	A	0.66	0.018	5.44E-11	0.0	0.976	0.017	3.77E-06	0.017	3.77E-06
rs12772375	10	104082688	T	0.40	-0.015	1.56E-08	14.1	0.178	-0.016	1.31E-05	-0.016	1.31E-05
rs7945718	11	12748819	A	0.63	0.015	1.54E-08	0.0	0.638	0.012	9.47E-04	0.012	9.47E-04
rs7955289	12	14653667	A	0.61	0.017	4.49E-10	0.0	0.816	0.015	3.31E-05	0.015	3.31E-05

rs2456973	12	56416928	A	0.67	-0.020	1.06E-12	10.7	0.239	-0.020	1.26E-07	-0.020	1.26E-07
rs7131944	12	92159557	A	0.62	0.015	9.02E-09	9.6	0.262	0.018	4.10E-07	0.018	4.10E-07
rs572016	12	121279083	A	0.51	0.014	3.46E-08	0.0	0.587	0.011	1.83E-03	0.011	1.83E-03
rs7306755	12	123767929	A	0.21	0.023	1.26E-12	0.0	0.668	0.027	1.76E-10	0.027	1.76E-10
rs9537821	13	58402771	A	0.72	0.024	1.50E-16	0.0	0.993	0.030	1.48E-14	0.030	1.48E-14
rs1043209	14	23373986	A	0.61	0.018	1.82E-11	0.0	0.721	0.018	3.15E-07	0.018	3.15E-07
rs8005528	14	27098611	A	0.75	-0.018	7.19E-09	0.0	0.631	-0.017	4.58E-05	-0.017	4.58E-05
rs17119973	14	84913111	A	0.26	-0.019	3.55E-10	30.4	0.013	-0.014	3.23E-04	-0.014	3.23E-04
rs192818565	17	43991515	T	0.81	0.025	1.47E-12	16.0	0.174	0.025	1.88E-07	0.025	1.88E-07
rs12969294	18	35186122	A	0.34	-0.016	7.24E-09	7.1	0.317	-0.015	8.24E-05	-0.015	8.24E-05
rs2837992	21	42620520	T	0.39	0.015	3.80E-08	0.0	0.565	0.015	3.41E-05	0.015	3.41E-05
rs165633	22	29880773	A	0.74	-0.018	2.86E-09	8.6	0.287	-0.015	1.92E-04	-0.015	1.92E-04

Supplemental Table 1. Univariable regression models stratified by sex, adjusted for age and genetic risk score of myopia (using generalized estimation equations). Data from the Gutenberg Health Study (2012-2017).

	**Year of Education**		
**Female participants:**			
	Estimate	95%-CI	p
Spherical Equivalent	-0.10	[-0.13—0.06]	<0.001
Axial Length	0.06	[0.04-0.08]	<0.001
Corneal Curvature	0.01	[-0.00-0.01]	0.01
Anterior chamber depth	0.01	[-0.00-0.01]	0.01
Lens thickness	-0.0002	[-0.01-0.00]	0.08
**Male participants:**			
Spherical Equivalent	-0.11	[-0.14- -0.08]	<0.001
Axial Length	0.06	[0.04-0.07]	<0.001
Corneal Curvature	-0.00001	[-0.00-0.00]	0.96
Anterior chamber depth	0.009	[-0.00-0.00]	0.001
Lens thickness	-0.003	[-0.01-0.00]	0.10
